# Heparanase Levels Are Elevated in the Urine and Plasma of Type 2 Diabetes Patients and Associate with Blood Glucose Levels

**DOI:** 10.1371/journal.pone.0017312

**Published:** 2011-02-22

**Authors:** Itay Shafat, Neta Ilan, Samih Zoabi, Israel Vlodavsky, Farid Nakhoul

**Affiliations:** 1 Cancer and Vascular Biology Research Center, The Bruce Rappaport Faculty of Medicine, Technion, Haifa, Israel; 2 Clinical Transplantation Unit, Rambam Health Care Campus, Haifa, Israel; 3 Department of Nephrology, Rambam Health Care Campus, Haifa, Israel; Mayo Clinic College of Medicine, United States of America

## Abstract

Heparanase is an endoglycosidase that specifically cleaves heparan sulfate side chains of heparan sulfate proteoglycans. Utilizing an ELISA method capable of detection and quantification of heparanase, we examined heparanase levels in the plasma and urine of a cohort of 29 patients diagnosed with type 2 diabetes mellitus (T2DM), 14 T2DM patients who underwent kidney transplantation, and 47 healthy volunteers. We provide evidence that heparanase levels in the urine of T2DM patients are markedly elevated compared to healthy controls (1162±181 vs. 156±29.6 pg/ml for T2DM and healthy controls, respectively), increase that is statistically highly significant (P<0.0001). Notably, heparanase levels were appreciably decreased in the urine of T2DM patients who underwent kidney transplantation, albeit remained still higher than healthy individuals (P<0.0001). Increased heparanase levels were also found in the plasma of T2DM patients. Importantly, urine heparanase was associated with elevated blood glucose levels, implying that glucose mediates heparanase upregulation and secretion into the urine and blood. Utilizing an *in vitro* system, we show that insulin stimulates heparanase secretion by kidney 293 cells, and even higher secretion is observed when insulin is added to cells maintained under high glucose conditions. These results provide evidence for a significant involvement of heparanase in diabetic complications.

## Introduction

Heparanase is an endoglycosidase which cleaves heparan sulfate (HS) side chains at a limited number of sites, activity that is strongly implicated in cell dissemination associated with tumor metastasis, inflammation and angiogenesis [1]–[4]. Utilizing an ELISA method capable of detection and quantification of heparanase [5], we have recently reported that heparanase levels are elevated in the plasma of pediatric cancer patients, correlating with their response to anticancer treatment [6]. Similarly, elevated levels of heparanase were measured in the urine of bladder cancer patients, associating with disease progression [7].

Emerging evidence indicate that heparanase is also engaged in diabetes and related complications, primarily kidney dysfunction [8], [9]. Loss of HS was observed in several experimental and human glomerulopathies, including diabetic nephropathy, minimal change disease, and membranous glomerulopathy [8], where a decrease in HS inversely correlates with proteinuria [10]–[13]. Decreased content of HS noted in the glomerular capillary barrier is attributed, in part, to over-expression of heparanase and, consequently, alteration of the glomerular basement membrane and its filtration characteristics [14]–[22]. Applying our ELISA method, we have previously demonstrated elevated levels of heparanase in the urine of a small group of diabetic patients, elevation that was confirmed by increased heparanase enzymatic activity in the urine of these patients [5], [23]. Here, we examined heparanase levels in the plasma and urine of patients with type 2 diabetes mellitus (T2DM), T2DM patients who underwent kidney transplantation and control healthy volunteers. Heparanase levels were then correlated with clinical and pathological data. We provide evidence that heparanase levels are elevated in the urine and plasma of T2DM patients and are reduced following kidney transplantation. Notably, glucose levels in the blood correlated with increased heparanase levels in the urine (P = 0.0001) and plasma (p = 0.003). Utilizing an *in vitro* system, we show that insulin stimulates heparanase secretion by kidney 293 cells, and even higher secretion is observed when insulin was added to cells maintained under high glucose conditions. The results imply that heparanase is engaged in diabetes and related complications and, thus, may serve as a diagnostic marker and drug target for T2DM.

## Materials and Methods

### Experimental design

The study included 47 healthy volunteers (controls) and 43 T2DM patients, 14 of which underwent kidney transplantation at the Rambam Health Care Campus (Haifa, Israel). The study was approved by the institutional review board. Clinical records included demographic and clinical data (i.e., renal function, plasma levels of glucose, creatinine, HbA1C, and cholesterol), treatment modalities and transplantation procedure. T2DM patients were selected at the outpatient nephrology clinic and characterized by albuminuria and near normal eGFR (i.e., 60–70 ml/min) typical of stage I-II diabetic kidney disease. Patients with early stage chronic kidney disease (i.e., close to normal creatinine levels) were selected as these patients are the ones likely to benefit from heparanase-based treatment modalities. Patients with stage V kidney disease who were subjected to kidney transplantation as replacement therapy due to diabetic nephropathy were selected for the study.

### Sample collection

A total of 3 ml peripheral blood was collected in EDTA-containing tubes, centrifuged (1500 g, 15 min at 4°C), and the supernatant was collected, aliquoted and kept at -80°C until use. Urine was collected, centrifuged at 1500 g for 10 min to remove cells and cell debris, and the supernatant was kept at −80^o^C until analyzed. All samples were thawed once.

### ELISA

Quantification of heparanase by ELISA method was carried essentially as described [5]. Briefly, wells of microtiter plates were coated (18 h, 4°C) with 1 µg/ml of 1E1 anti-heparanase monoclonal antibody (mAb) in 50 µl of coating buffer (0.05 M Na_2_CO_3_, 0.05 M NaHCO_3_, pH 9.6). After blocking (1% BSA in PBS, 1 h at 37°C), plasma (100 µl, diluted 1∶1 with 0.5% BSA) or urine (200 µl) samples were loaded in duplicates and incubated at room temperature for 2 h, followed by the addition of anti-heparanase polyclonal antibody 1453 (1 µg/ml) for additional 2 h. HRP-conjugated goat anti-rabbit IgG (1∶20,000; Jackson ImmunoResearch, West Grove, PA) in blocking buffer was added (1 h) and the reaction was visualized by the addition of 100 µl chromogenic substrate (TMB) for 30 min. The reaction was stopped with 100 µl H_2_SO_4_ and absorbance at 450 nm was measured with reduction at 630 nm using ELISA plate reader. Plates were washed five times with washing buffer (PBS, pH 7.4, containing 0.1% Tween 20) after each step. As a reference for quantification, a standard curve was established by a serial dilution of recombinant 8+50 (GS3) active heparanase enzyme (390 pg/ml–25 ng/ml) [5]–[7]. The 1E1 mAb preferentially recognizes the 8+50 kDa active enzyme vs. the 65 kDa proenzyme [5].

### Cell culture, heparanase secretion and immunoblotting

Heparanase-transfected human embryonic kidney 293 cells stably expressing heparanase and control cells transfected with an empty vector (Vo) have been described previously [24]. Heparanase cDNA cloned in pcDNA3 vector was used to direct high levels of expression in mammalian cells, driven by a strong viral (CMV) promoter. While this vector is not appropriate for the study of gene transcription, it is most suitable for post-transcriptional and secretion studies. Cells were grown to confluence followed by incubation for 20 h in serum-free medium. The medium was then replaced by fresh serum-free medium and the cells were incubated without or with the indicated concentration of insulin (representing the range of insulin oscillation [25]) for additional 2 h. In other experiments, cells were grown in normal growth medium [i.e., 0.45% (25 mM) glucose] or high glucose conditions [3% (167 mM) glucose] without or with insulin. Conditioned medium was collected and applied onto ^35^S-labeld ECM-coated dishes to evaluate heparanase enzymatic activity (see below) or, following enrichment on heparin-Sepharose beads, were subjected to immunoblotting, as described [24].

### Heparanase activity

Preparation of sulfate labeled ECM-coated dishes and determination of heparanase activity were performed as described in detail elsewhere [24], [26]. Briefly, medium conditioned by 293 cells (5×10^5^–2×10^6^) was adjusted to pH 6.0, applied into 35 mm dishes coated with ^35^S-labeled ECM and incubated for 8 h at 37°C. The incubation medium containing sulfate-labeled degradation fragments was subjected to gel filtration on a Sepharose CL-6B column. Fractions (0.2 ml) were eluted with PBS and their radioactivity counted in a β-scintillation counter. Degradation fragments of HS side chains were eluted at 0.5< Kav<0.8 (peak II, fractions 10–30). Nearly intact HSPG was eluted just after the Vo (Kav<0.2, peak I, fractions 3–8) [24], [26].

### Statistical analysis

Values are expressed as means±SE or as medians with ranges. Comparison of heparanase levels between the different groups was evaluated using the non-parametric Mann–Whitney U test. The non-parametric Spearman's rank test was used to calculate correlation between the different variables. A value of P<0.05 was considered significant. GraphPad Prism 4.0 (GraphPad Software Inc., San Diego, CA, USA) was used for data analysis.

### Ethics

The study was approved by the Institution (Rambam Health Care Campus) Review Board. A written informed consent was obtained from each participant.

## Results

### Heparanase levels are elevated in the urine and plasma of T2DM patients

Heparanase levels were quantified in 29 type 2 diabetic patients (T2DM), 14 T2DM patients who underwent kidney transplantation, and 47 healthy volunteers utilizing an ELISA method specific for human heparanase [5]. Demographic data is summarized in Table 1. The control group (30 male, 17 female, age 46±10) included individuals with normal blood glucose levels (78.9±1.1 mg/dl; 4.4 mM), blood pressure (120/80 mmHg), and kidney function (i.e., creatinine levels ≤1 mg/dl). In T2DM patients (14 female, 15 male; age 56.2±12; average length of disease = 14.3 years), plasma creatinine level was 1.25±0.6 mg/dl, blood glucose was 148.5±18.3 mg/dl (8.3 mM) and HbA1c was 5.5–7.5%. Nine patients were treated with insulin while all the rest were treated with oral anti-glycemic drugs such as Metformin and Sulfanilurea (Glibetic). In transplanted patients (3 female, 11 male; age 58.4±12.6), plasma creatinine was 1.38±0.75 mg/dl, glucose was 135.6±38.2 mg/dl (7.5 mM) and HbA1C was over 7%. We selected patients that were transplanted 5.2 years in average prior to blood and urine sampling, bearing functioning kidney and exhibiting creatinine levels close to normal (Table 1).

**Table 1:** Demographic description of enrolled individuals.

Urine heparanase (pg/ml)	Control (n)	T2DM (n)	Transplanted (n)
Male	169±41 (26)	1279±270 (15)	394±95 (11)
Female	141±42 (21)	1037±244 (14)	516±100 (3)
Average ± SE	156±29	1162±181	424±76
Total	47	29	14

Plasma heparanase (pg/ml)	Control (n)	T2DM (n)	Transplanted (n)
Male	110±24 (18)	225±40 (6)	148±45 (11)
Female	125±21 (18)	448±110 (8)	372±97 (3)
Average ± SE	117±16	352±70	196±46
Total	36	14	14

**Figure 1:**
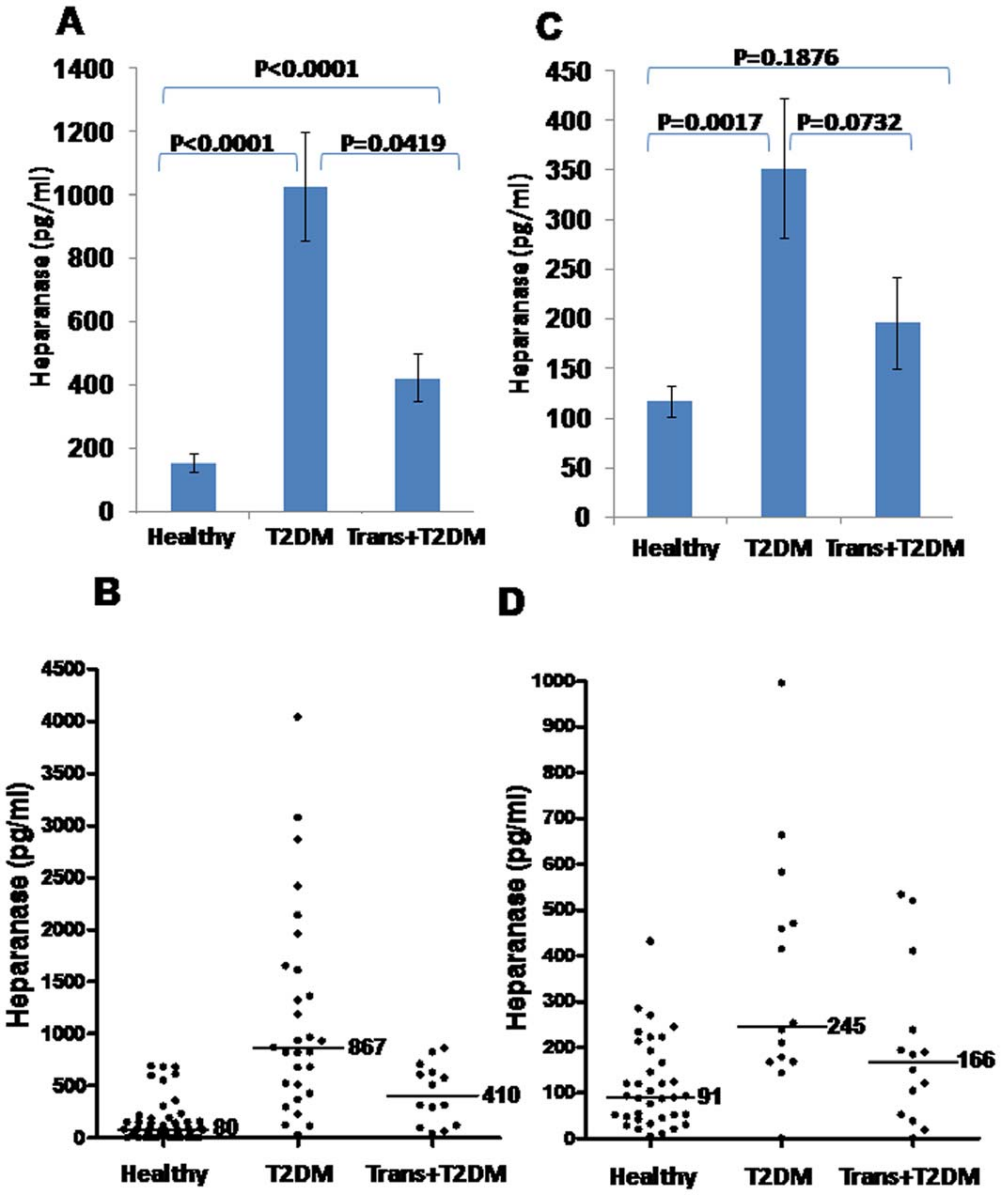
Determination of heparanase levels in urine (A, B) and plasma (C, D) of individuals from the study groups. Shown are average (±SE; A, C) and median (B, D) values quantified by an ELISA method, as described under ‘Materials and Methods’.

Heparanase levels in the urine of T2DM patients were markedly elevated compared to healthy controls (1162±181 vs. 156±29.6 pg/ml for T2DM and healthy controls, respectively; Table 2, Fig. 1A), increase that is statistically highly significance (P<0.0001). Heparanase levels were appreciably decreased in the urine of T2DM patients who underwent kidney transplantation (1162±181 vs. 424±76 pg/ml for T2DM and transplanted patients, respectively; Table 2, Fig. 1A; p = 0.04), albeit still higher than healthy individuals (P<0.0001). A more representative view of heparanase levels in the urine of T2DM and transplanted patients is obtained by plotting median, rather than average values (Fig. 1B). While a median value of 80 pg/ml was calculated for healthy individuals (control), median values of 867 and 410 pg/ml were calculated for T2DM and transplanted patients, respectively (Fig. 1B). These results suggest that urinary heparanase originates primarily from diabetes-related nephropathy.

**Table 2:** Heparanase levels in the urine and plasma of healthy volunteers (control), type 2 diabetic patients (T2DM) and T2DM patients who underwent kidney transplantation.

	Healthy control	T2DM	T2DM+ transplantation
N	47	29	14
% Male	64	52	78
Age (years)	46.1±10	56.2±12	58.4±12.6
Blood creatinine (mg/dl)	1±0.1	1.25±0.6	1.38±0.75
eGFR (MDRD, ml/min)	75.7±2.7	62.03±4.7	68.64±8.6
Glucose, mg/dl (mM)	78.9±1.1 (4.4)	148.5±18.3 (8.3)	135.6±38.2 (7.5)

Increased heparanase levels were also found in the plasma of T2DM patients (352±70.4 vs. 117±16 pg/ml for T2DM and control, respectively; P = 0.001) (Table 2, Fig. 1C), as also reflected by plotting median values (Fig. 1D). Heparanase levels in the plasma of T2DM patients were decreased after kidney transplantation (352±70 pg/ml vs. 196±46), though only approaching statistical significance (P = 0.07; Fig. 1C), likely due to the low number of patients in this group. Thus, in T2DM, elevated amounts of heparanase are found in the circulation, spread systemically and may affect distant organs, tissues, and cells.

**Figure 2:**
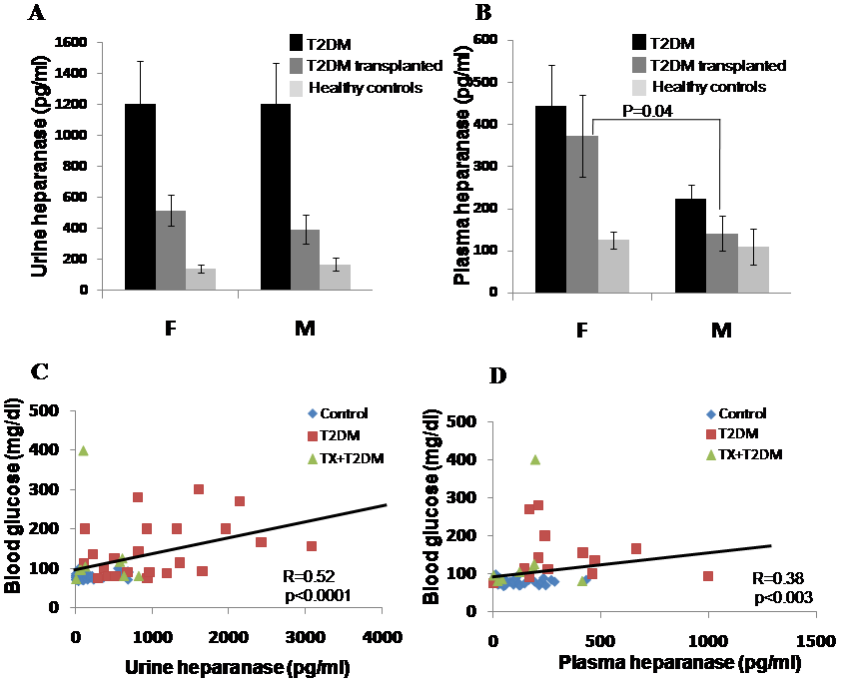
Association between blood glucose and heparanase levels. **A, B**. Urine and plasma heparanase levels presented according to gender. Shown are average (±SE) values of heparanase levels in urine (A) and plasma (B) quantified by ELISA in males (M) and females (F) of healthy volunteers (control), T2DM patients and T2DM patients who underwent kidney transplantation. **C, D**. Association between urine and plasma heparanase and blood glucose levels. Heparanase levels in plasma and urine were correlated with blood glucose levels using the non-parametric Spearman's rank test. Heparanase levels in the urine were found to correlate with blood glucose (**C**; r = 0.52, p = 0.0001); blood glucose was also associated with plasma heparanase (**D**; r = 0.38, p = 0.003).

We have previously reported that heparanase enzymatic activity is preferentially elevated in urine collected from type 1 diabetic females vs. males [23]. We therefore analyzed heparanase levels according to gender (Fig. 2A, B). No differences in heparanase levels were observed in the urine of T2DM females and males (Fig. 2A; Table 2). Interestingly, higher levels of heparanase were quantified in the plasma of T2DM females compared to males (448±110 and 225±40 for females and males, respectively; Fig. 2B, Table 2). This difference only approached statistical significance (p = 0.1). Accordingly, higher levels of plasma heparanase were found in female patients who underwent kidney transplantation compared with males (372±9 and 148±45, respectively; Fig. 2B, Table 2), differences that are statistically significance (p = 0.04). Elevated heparanase levels in the plasma of females vs. males may be due to estrogen shown to stimulate heparanase expression by estrogen receptor-positive cells [27], [28]. We next examined the association between heparanase levels in the plasma and urine of patients and their blood glucose levels. Notably, blood glucose was significantly associated with urine (r = 0.52, P = 0.0001; Fig. 2C), and plasma heparanase (r = 0.38, P = 0.003; Fig. 2D).

### Insulin cooperates with glucose in stimulating heparanase secretion

**Figure 3:**
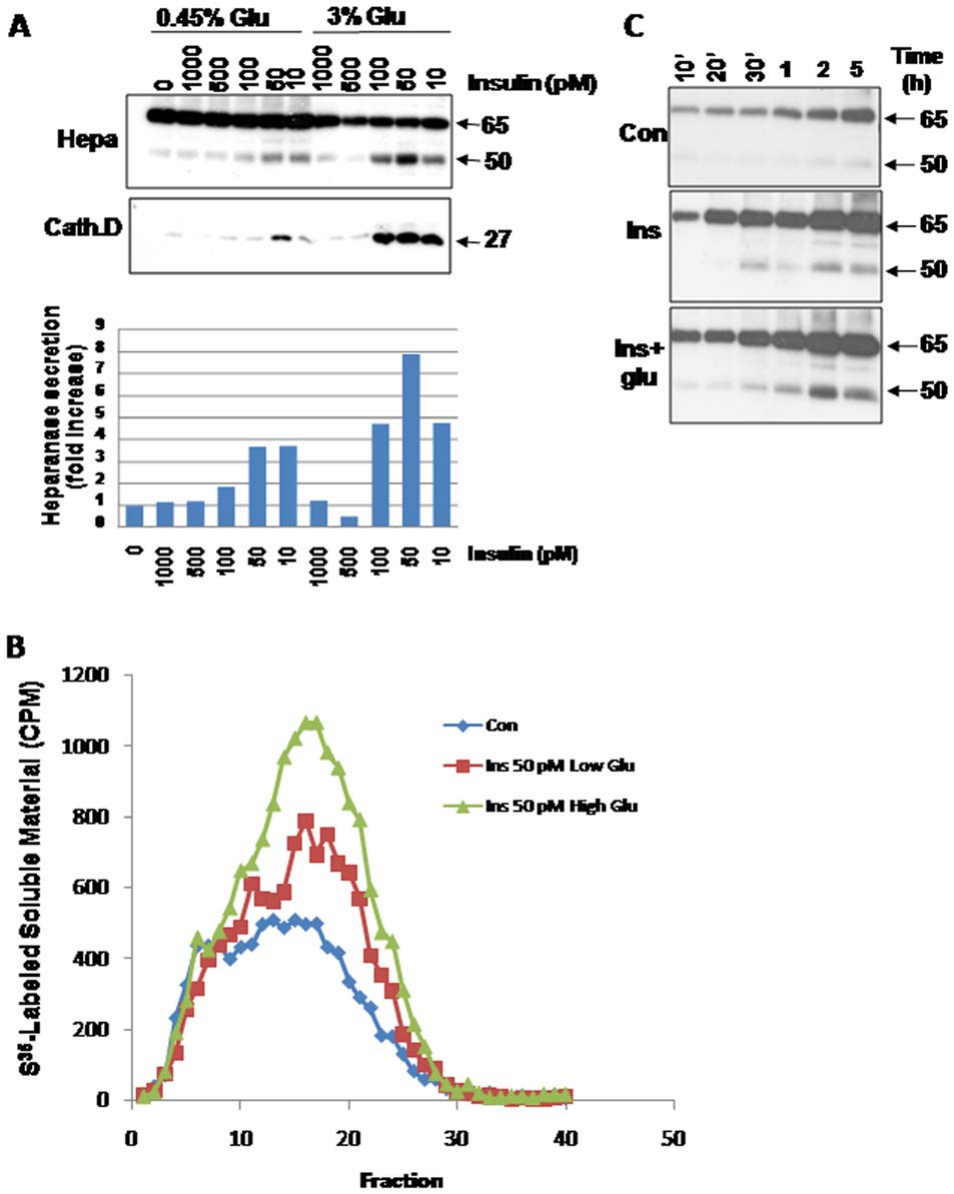
Insulin cooperates with glucose to stimulate secretion of enzymatically active heparanase. **A**. Immunoblotting. Heparanase-transfected 293 cells were cultured under normal (0.45%) or high (3%) glucose conditions in serum free medium for 20 h. Cells were left untreated (0) or incubated with the indicated concentration of insulin for 2 h. Cell conditioned medium (1 ml) was then collected, and TCA-precipitates were subjected to immunoblotting applying anti-heparanase (upper panel) and anti-cathepsin D (second panel) antibodies. Densitometry analysis of the active 50 kDa heparanase is shown in the lower panel. **B**. Heparanase enzymatic activity. Corresponding medium samples of untreated cells (control; ♦) or cells treated with insulin (50 pM) under low (▪) or high (▴) glucose were applied onto 35 mm dishes coated with ^35^S-labeled ECM for 20 h. The medium was then collected and sulfate labeled HS degradation fragments were analyzed by gel filtration, as described under "Materials and Methods". **C.** Kinetics. Heparanase transfected 293 cells were grown under serum-free conditions and left untreated (Con) or stimulated with insulin under low (0.45%; Ins) or high (3%; Ins+glu) glucose levels. At the time indicated, conditioned medium was collected and subjected to immunoblotting applying anti-heparanase antibody.

**Figure 4:**
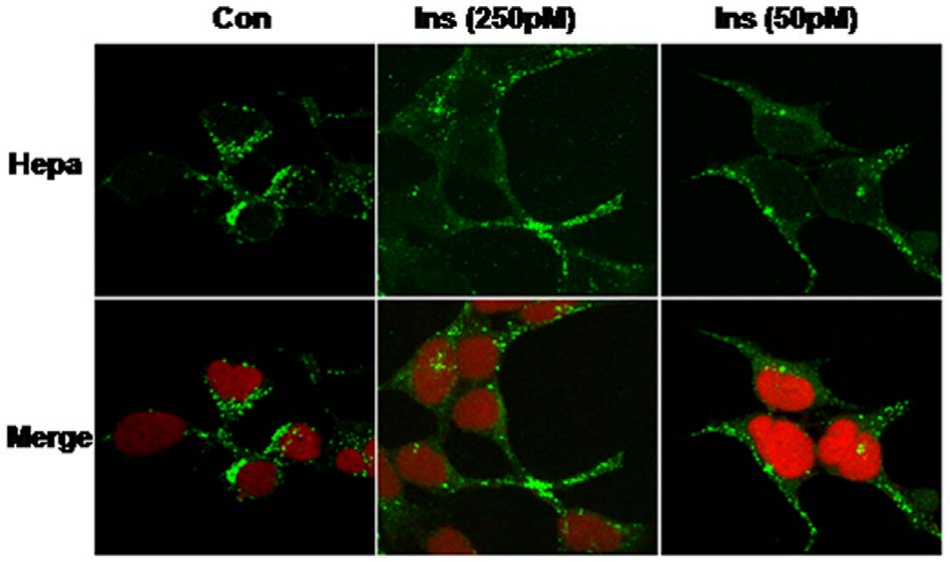
Immunofluorescent staining. Heparanase transfected 293 cells were left untreated as control (Con) or stimulated with insulin (250 and 50 pM) for 2 h. Cells were then fixed, stained with monoclonal anti-heparanase antibody (upper panel, green) and examined by confocal microscopy. Merged images with nuclear counterstaining (red) are shown in the lower panels. Note more diffused heparanase-positive vesicles in response to insulin stimulation.

While the 65 kDa pro-heparanase includes a signal peptide (Met^1^-Ala^35^) and is readily secreted, secretion of active (8+50 kDa) heparanase is tightly regulated [2], [24]. The association between blood glucose and urine heparanase levels led us to hypothesize that glucose, insulin or combination of the two, stimulate heparanase secretion. In order to examine this possibility, heparanase-transfected human embryonic kidneys (HEK)-293 cells were left untreated or stimulated with increasing concentrations of insulin. Conditioned medium was collected after 2 h and subjected to immunoblotting applying anti-heparanase antibody. No significant change in the levels of latent 65 kDa heparanase was observed (Fig. 3A). In contrast, insulin stimulated secretion of the active 50 kDa heparanase (Fig. 3A). Interestingly, low concentrations of insulin appeared more effective than high doses (10, 50 vs. ≥100 pM; Fig. 3A). Densitometry analysis revealed a nearly 4-fold increase in heparanase (50 kDa) secretion in response to low vs. high insulin concentrations (Fig. 3A, third panel). Accordingly, heparanase activity was markedly increased in the conditioned medium of cells stimulated with insulin at low concentrations (not shown). Even higher stimulation of heparanase secretion was observed in cells grown under high glucose levels (3%; 167 mM) and treated with low concentrations (10–100 pM) of insulin (Fig. 3A). Thus, while insulin stimulated heparanase secretion by ∼4-fold in cells grown under normal glucose levels (0.45%; 25 mM) (Fig. 3A, third panel), nearly 8-fold increase in heparanase secretion was observed in cells grown in 3% (167 mM) glucose and incubated with insulin (Fig. 3A). Accordingly, a higher heparanase activity was measured in medium conditioned by cells stimulated with insulin under high glucose (Fig. 3B). Co-operation between glucose and insulin in stimulating both latent (65 kDa) and active (50 kDa) heparanase secretion was similarly demonstrated by kinetics studies (Fig. 3C). Evidently, the combination of insulin and glucose did not accelerate heparanase secretion but rather increased its magnitude (Fig. 3C). The secreted active 50 kDa heparanase most likely originates from lysosomes. This is concluded from a parallel enhanced secretion of the active (27 kDa) form of cathepsin D, a lysosome-resident protease (Fig. 3A, second panel), and the re-distribution of heparanase-containing vesicles in cells treated with insulin (Fig. 4). Thus, while in un-stimulated cells heparanase was mainly detected in endocytic vesicles residing perinuclearly (Fig. 4, Con), heparanase-positive vesicles appeared redistributed towards the cell periphery following insulin treatment (Fig. 4, Ins). This pattern of vesicles relocation is in agreement with previous characterization of mechanisms that facilitate secretion of active heparanase [24].

## Discussion

While heparanase function in tumor biology is well documented [2], [4], [29] and heparanase inhibitory compounds are being developed as anti-cancer drugs [4], [30]–[35], emerging evidence indicate that heparanase is also engaged in several other pathological disorders. Of increasing importance is the apparent role of heparanase in diabetes and related complications, primarily diabetic nephropathy [8], [9]. Expression of heparanase is up-regulated in the course of diabetic nephropathy and other kidney diseases [16], [17], possibly destructing the permselective properties of HS. Heparanase activity correlates with damage and dysfunction of the glomerular basement membrane, leading in many cases to proteinuria [9], [22], [23]. Results presented in this study add another layer of evidence for the causal involvement of heparanase in diabetes. Clearly, heparanase levels are significantly elevated in the urine of type 2 diabetic patients (Fig. 1A, B). This is in agreement with previous publications demonstrating increased heparanase activity [23] and protein levels [18] in the urine of a small group of diabetes patients. Decreased urine heparanase levels in diabetic patients who underwent kidney transplantation suggest that urinary heparanase originates primarily from diabetes-associated kidney failure. Indeed, immunohistochemical analysis showed that heparanase is expressed in tubular epithelial cells but not in glomeruli of normal kidney, while its expression is upregulated in the glomeruli and tubular epithelial cells in kidneys with diabetic nephropathy [17], [18]. It should be kept in mind that transplanted patients are treated with immunosuppressive drugs, in addition to diabetes-related medication, which may affect heparanase secretion.

The observed association with blood glucose levels (Fig. 2C) may imply that glucose is involved in heparanase regulation. Previously, glucose has been shown to stimulate heparanase gene expression in rat glomerular epithelial cells and human embryonic kidney 293 cells, associating with decreased levels of surface HS [18]. Our results suggest that, in addition, glucose stimulates heparanase secretion. This is best demonstrated by enhanced heparanase secretion from cultured kidney-derived cells (293) stably expressing heparanase in response to insulin, and even more so when insulin is combined with high glucose levels (Fig. 3).

Importantly, elevated levels of heparanase were also found in the plasma of diabetes patients (Fig. 1C, D), ultimately leading to systemic spread of heparanase, possibly affecting distant organs, tissues and cells. In diabetic patients, vascular endothelial cells are under continuous exposure to high glucose and insulin levels. In previous studies, glucose (25 mM) was found to stimulate secretion of heparanase by coronary artery endothelial cells, resulting in decreased intracellular content of heparanase [36]. Furthermore, high levels of glucose stimulated redistribution of heparanase-positive lysosomal vesicles in endothelial cells [36], resembling the effect of insulin on kidney 293 cells (Fig. 4). Interestingly, exposing endothelial cells to high glucose was accompanied by a rapid increase in extracellular ATP [36] which apparently mediates the enhanced secretion of heparanase by glucose [36]. This result is in perfect agreement with previous reports implicating adenosine, ATP, and the P2Y receptors in heparanase secretion by tumor-derived and kidney 293 cells [24]. Thus, elevated levels of heparanase found in the plasma of diabetic patients may originate from enhanced secretion of heparanase by kidney and endothelial cells. The latter may account for the relatively high levels of heparanase determined in the plasma of patients following kidney transplantation (Fig. 1C, D; Table 2), where glucose is still present at high amounts and likely affects the vascular endothelium while kidney function is relatively normal. Continuous exposure of the vascular endothelium to heparanase may have severe outcome. It has been shown, for example, that heparanase over-expression leads to arterial thickening and reduced mechanical integrity [37]. Moreover, heparanase over-expression enhances neointimal thickening due to increased proliferation and recruitment of macrophages following stent implementation [37] whereas neutralizing anti-heparanase antibodies reduced neointima formation in a rat carotid balloon injury model [38]. Diabetes is also associated with development of prothrombotic conditions [39]. Accumulating evidence suggest that heparanase functions as a pro-coagulation mediator, enhancing expression of tissue factor and generation of factor Xa, two critical components in blood coagulation [40]–[42] thus providing another mode by which heparanase affects the vasculature. Collectively, these results imply that continuous exposure to high levels of heparanase in the circulation may contribute to the development of diabetic-associated vasculopathies.

While glucose has been shown to be engaged in heparanase regulation [36], [43], the ability of insulin to stimulate heparanase secretion is shown here for the first time. Insulin and insulin-like growth factor are key regulators of energy metabolism and growth. In addition, considerable evidence indicates that these hormones and the signal transduction networks they regulate have important roles in neoplasia [44]. Hence, increased insulin levels seen in association with type 2 diabetes might lead to aggressive tumor behavior [44]. Given the well documented role of heparanase in tumor metastasis and angiogenesis [2], [4], [45], it is plausible that insulin, with or without glucose, stimulates secretion of active heparanase by tumor cells, thus accelerating tumor progression. Studies examining this possibility are currently underway.

Taken together, the results of this study support and further extend the causal involvement of heparanase in diabetes and the associated complications, and provide for the first time clinical association between blood glucose and heparanase secretion. Decreased albumin excretion following administration of low molecular weight heparin and related compounds, including oral sulodexide [46]–[48] is attributed, in part, to the potent heparanase-inhibiting capacity of heparin mimicking compounds [4], [30], [49], [50]. These and related compounds are thus hoped to develop into new treatment option for patients with diabetic nephropathy, diabetes-related vascular and neurological pathologies, and cancer.
